# Estimating herbaceous aboveground biomass in Sahelian rangelands using Structure from Motion data collected on the ground and by UAV

**DOI:** 10.1002/ece3.8867

**Published:** 2022-05-01

**Authors:** Simon Taugourdeau, Antoine Diedhiou, Cofélas Fassinou, Marina Bossoukpe, Ousmane Diatta, Ange N’Goran, Alain Auderbert, Ousmane Ndiaye, Abdoul Aziz Diouf, Torbern Tagesson, Rasmus Fensholt, Emile Faye

**Affiliations:** ^1^ CIRAD UMR SELMET‐ PPZS Dakar Senegal; ^2^ UMR SELMET CIRAD, INRA Institut Agro Univ Montpellier Montpellier France; ^3^ 89230 Departement Biologie végétale ‐ PPZS UCAD Dakar Senegal; ^4^ ISRA CRZ (Centre de Recherches Zootechniques) Dahra‐PPZS Dahra Djoloff Senegal; ^5^ CIRAD, INRAE UMR AGAP Institut Univ Montpellier Montpellier France; ^6^ Centre de Suivi Ecologique ‐ PPZS Dakar Sénégal; ^7^ 5193 Department of Physical Geography and Ecosystem Science Lund University Lund Sweden; ^8^ Department of Geosciences and Natural Resource Management University of Copenhagen Copenhagen Denmark; ^9^ UPR Hortsys CIRAD Univ Montpellier Montpellier France

**Keywords:** 3D model, herbaceous aboveground biomass, savannah ecosystem, Senegal, Unmanned Aerial Vehicle, vegetation index

## Abstract

Herbaceous aboveground biomass (HAB) is a key indicator of grassland vegetation and indirect estimation tools, such as remote sensing imagery, increase the potential for covering larger areas in a timely and cost‐efficient way. Structure from Motion (SfM) is an image analysis process that can create a variety of 3D spatial models as well as 2D orthomosaics from a set of images. Computed from Unmanned Aerial Vehicle (UAV) and ground camera measurements, the SfM potential to estimate the herbaceous aboveground biomass in Sahelian rangelands was tested in this study. Both UAV and ground camera recordings were used at three different scales: temporal, landscape, and national (across Senegal). All images were processed using PIX4D software (photogrammetry software) and were used to extract vegetation indices and heights. A random forest algorithm was used to estimate the HAB and the average estimation errors were around 150 g m^−^² for fresh mass (20% relative error) and 60 g m^−^² for dry mass (around 25% error). A comparison between different datasets revealed that the estimates based on camera data were slightly more accurate than those from UAV data. It was also found that combining datasets across scales for the same type of tool (UAV or camera) could be a useful option for monitoring HAB in Sahelian rangelands or in other grassy ecosystems.

## Graphical abstract

We tested UAV and Ground camera photogrammetry for the evaluation of herbaceous biomass of Sahelian rangeland. We show that both UAV and ground camera can be used for the evaluation of the biomass during a season and across different spatial scales. Our work shows the possibility of the intercalibration between different datasets.

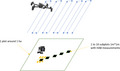

## 1 INTRODUCTION

Grass or herbaceous vegetation is present in most ecosystems on earth, and herbaceous aboveground biomass (HAB) has been used as an indicator of ecosystem productivity and functioning in a plethora of studies in relation to biodiversity‐ecosystem functioning relationships (Hector et al., 1999). HAB is also the main source of feed for many wild and domestic animals, and is used in many studies to assess feed availability (Hiernaux et al., 2015; Ickowicz, 1995).

HAB is usually collected by destructive sampling (removal of vegetation), which is a laborious work method, so the development of indirect sampling using remote sensing methods has been under continuous development for several decades (Reinermann et al., 2020). Many tools are based on measuring the spectral characteristics of vegetation. Such measurements are normally used to calculate indices (proxies of vegetation) based on a combination of near‐infrared and visible reflectance of the vegetation (Bannari et al., 1995; Barbosa et al., 2019; Rouse et al., 1974). These indices are generally based on satellite remote sensing images, but can also be derived from images directly taken on the ground with a camera, or from near‐field remote sensing using Unmanned Aerial Vehicles (UAV) (Candiago et al., 2015; Cruzan et al., 2016; Grenzdörffer et al., 2008). Another tool for estimating HAB using remote sensing is the LiDAR technique (Schulze‐Brüninghoff et al., 2019). Therefore, the volume of herbaceous vegetation is subsequently derived from a LiDAR‐retrieved point cloud, which can be used as a proxy of HAB. Some studies have tested combining both the volume of the herbaceous vegetation and the spectral signature to estimate HAB (Schulze‐Brüninghoff et al., 2020).

Structure from Motion (SfM) is a photogrammetry process that can be used to create a variety of 3D spatial models and 2D orthomosaics from images (Schonberger & Frahm, 2016). The process relies on a set of images of the same object taken from different angular views, and the SfM produces a 3D point cloud of the object. This process is generally applied to UAV images and the point cloud from the set of images is used to produce an orthomosaic and a digital surface model (Frey et al., 2018). Color indices can be calculated based on the orthomosaic depending on the sensors used (multispectral or simple RGB sensors). Applying UAV imagery with the SfM process is widely used to estimate HAB from 3D‐based indices and vegetation indices (Aasen et al., 2015; Cunliffe et al., 2016; Kolarik et al., 2020; Possoch et al., 2016; Wijesingha et al., 2020). The SfM process can also be applied to imagery generated directly from sensors mounted on the ground. This process has been used to develop a 3D model of individual plants under greenhouse conditions, or directly in the field (An et al., 2017; Andújar et al., 2018; Bossoukpe et al., 2020; Cooper et al., 2017).

Most studies using the SfM process have been carried out in temperate grasslands and on a local scale (Lussem et al., 2018, 2019; Wijesingha et al., 2020). In this study, we assessed the applicability of the SfM methodology for estimating HAB in Senegalese savannah ecosystems including a large diversity of plant functional types. The objectives were to:

- Assess the applicability of the SfM methodology based on reflectance in the visible part of the spectrum (red‐green‐blue; RGB) for biomass monitoring.
- Compare the biomass estimated using the SfM methodology based on images collected both with UAVs and with a digital camera on the ground.
- Study whether the SfM methodology can be used to monitor HAB variability over the growing season, across a savannah landscape locally, and spatially across a diversity of plant functional types.
- Lastly, compare calibration carried out across the different datasets and calibration within each dataset in order to see whether existing datasets can be used for such calibration.

## 2 MATERIAL AND METHODS

**FIGURE 1:**
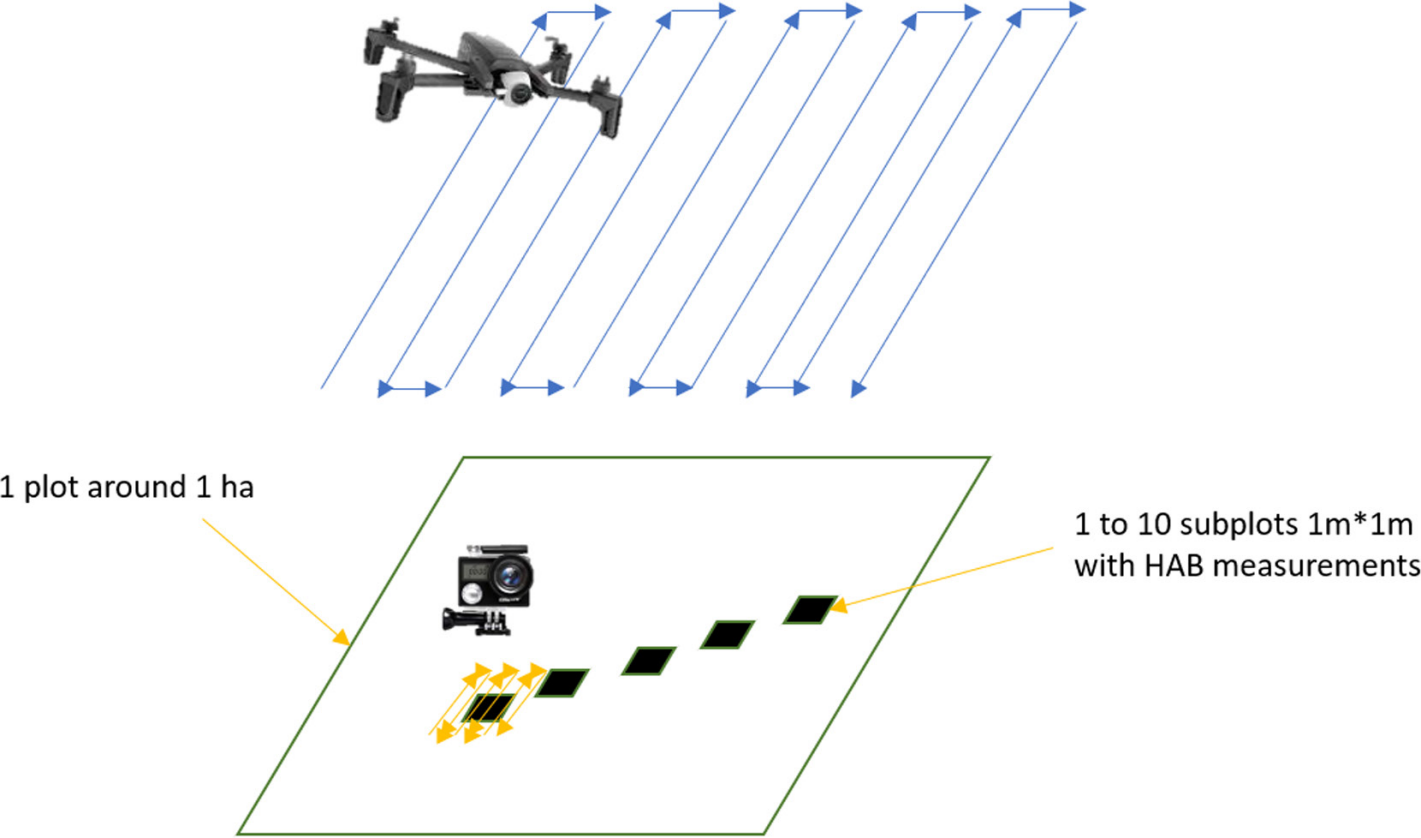
Schematic representation of the plot and the subplot(s). The positions of HAB are the black squares measuring 1 m by 1 m. For the Unmanned Aerial Vehicle (UAV), the images were taken over the entire plot. The camera images were taken over the subplots. There were from 1 to 10 subplots per plot

We collected RGB images using a camera mounted on an Unmanned Aerial Vehicle (UAV), and with a digital camera on the ground, combined with field measurements of herbaceous aboveground biomass. The different measurements (UAV, camera, and HAB) were carried out on plots of 1 ha. The UAV images covered all the plot. Within the plot, subplots of 1m by 1m were laid out. The camera plot and the HAB measurements were carried out on these subplots (Figure 1). The positions of the subplots were marked out with a wooden triangle painted in white, or with a plastic bag on the ground.

For the different datasets, we first present the position of the plot and the subplots and the dates of the different measurements.

Second, we detail the protocol for the image acquisitions and image analysis (Structure from Motion).

Lastly, we present the herbaceous biomass field measurements.

### 2.1 Design of the measurement setup

**FIGURE 2:**
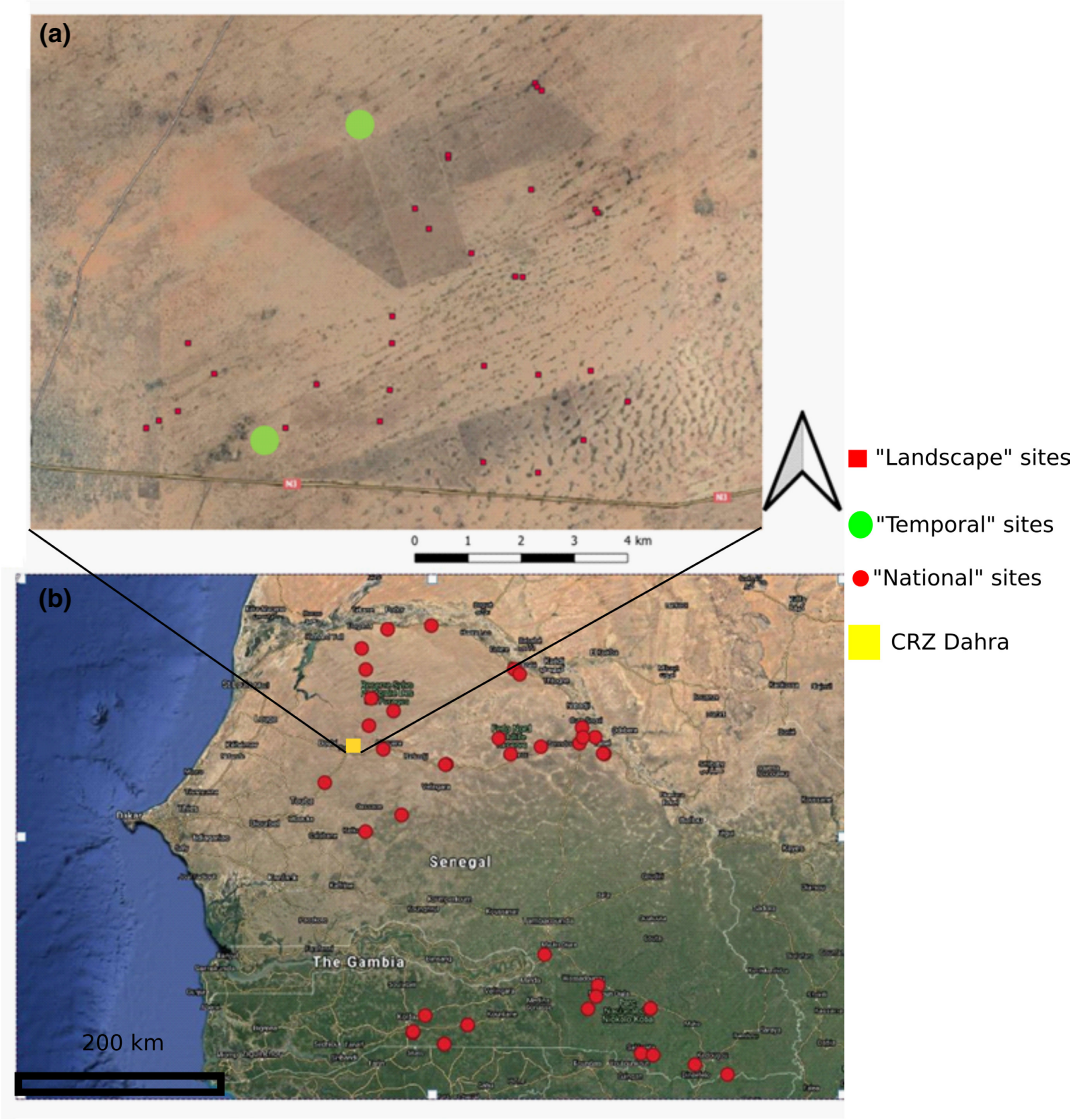
(a) Map of the CRZ (Centre de Recherches Zootechniques) near Dahra with the position of the different plots of the Landscape (red squares) and Temporal datasets (green dots). (b) Map of Senegal with the different plots of the National scale datasets (red dots). The yellow square represented the position of the CRZ Dahra (map a)

Data were collected on three spatio‐temporal scales for comparison:

1. Temporal scale: images and HAB data were collected at the same locations over the growing season (corresponding to the wet season between June and October) (Figure 2a).
2. Landscape scale: measurements were done within a 6,800‐ha area of enclosed savannah in northern Senegal (Figure 2a).
3. National scale: measurements were taken throughout Senegal (Figure 2b).

This design produced six datasets. Three datasets for the analysis of UAV imagery: Temporal UAV (TU), Landscape UAV (LU), and National UAV (NU); and three datasets for the digital camera on the ground: Temporal camera (TC), Landscape camera (LC), and National camera (NC).

#### 2.1.1 Temporal scale measurements

The temporal scale measurements were carried out at the *Centre de Recherches Zootechniques* in Dahra (Figure 1a), a research station in northern Senegal (15°21’ N, 15°28’ W) in the silvopastoral zone of the country referred to as the Ferlo region. The station covers an area of 6800 ha managed by ISRA (*Institut Sénégalais de Recherches Agricoles*). Mean annual rainfall over the last 50 years was 371.6 mm and the soils are mainly sandy (Ndiaye et al., 2014, 2015; Tagesson, Fensholt, Guiro, et al., 2015).

Images were taken from 1 m^2^ subplots every 10 days with the digital camera on the ground during the 2019 rainy season (end of July to October) and during the 2020 rainy season (July to October). In 2019, images were collected close to the Dahra research center office, and in 2020 they were collected around the Dahra flux tower site (Tagesson, Fensholt, Cropley, et al., 2015). Both years, the images were collected along two perpendicular 1 km transects (Diatta et al., 2021). Every 10 days, measurements were taken 1 m to the north of the last measurements (10 days earlier).

The UAV images were taken every two days during the 2020 rainy season, covering a one ha area. For the HAB measurements, three subplots of 1 m^2^ were sampled every time in relation to the distance from the center of a tree: one under the crown of the tree, one at the perimeter of the tree crown and one outside tree influence (at a greater distance than the height of the tree) to be compared with HAB measurements collected on the ground from destructive sampling (see description below). The position of the plot changed each time. In total, the measurements were taken around six different trees of two different species (*Balanites aegyptiaca* and *Vachellia tortilis*).

#### 2.1.2 Landscape scale measurement

For the landscape scale measurements, data were collected from 38 plots of 1‐ha close to the Dahra research station (Figure 2a, the red squares). These plots had already been used in several studies of vegetation dynamics and were selected to be representative of the diversity of vegetation types within the research station (Ndiaye et al., 2014, 2015; Raynal, 1964). Data were collected at the end of the rainy season (October 2018) when HAB was at its maximum. Images covering the full 1‐ha plots were collected with the camera mounted on the UAV. For HAB, 10 subplots of 1‐m² were measured along a 20 m line. The images with the digital camera on the ground were only taken for the first 1 m² subplot among the 10.

#### 2.1.3 National scale measurements

The data collected on a national scale were taken from 47 plots during two field campaigns (Figure 2b): one in the northern part of Senegal at the end September 2020 and the other in the southeastern part of Senegal in the middle of October 2020. The selection of plots was based on a combination of accessibility and diversity of vegetation. The soil was categorized into three soil types (13 sites with sandy soil, 16 with ferralitic soil, and 18 with clay soil) and ecosystem types were separated into four categories (13 steppes, 16 sparse savannah, 11 dense savannah, and 8 dense forests). The average annual rainfall for the 1981–2018 period in the different plots ranged from 221 mm to 468 mm for the northern part and 759 mm to 1246 mm for the southeastern part. We measured one to three 1‐m² subplots for each plot.

### 2.2 UAV and ground camera acquisition

#### 2.2.1 UAV flight plan

We used two low‐cost UAVs with integrated RGB (Red Green Blue) sensors.

For landscape scale data collection, the images were collected using a Dji Spark UAV with the litchi application (https://flylitchi.com) using automatic flight. The flight plan was six 100‐m transects 20 m apart at an altitude of 80 m and speed of 5 m s^−1^. Images were acquired with autofocus (ISO exposures were automatically adjusted) at two‐second intervals with an 80° angle of view throughout the flights. The frontal overlap was about 90% and the side overlap about 80%. The flights took place throughout the day between 8 am and 6 pm.

For temporal and national scale data collection, the UAV was a Parrot Anafi with a Pix4D capture application (drone flight planning app) using a double‐gridded flight plan that covered at least 1 ha. For data collection on the temporal scale, the height of the flight was 60 m with an overlap of 80% at low speed. All the flights were made early in the morning. Images were acquired in autofocus mode with an 80° angle.

For data collection on a national scale, we used the same double‐gridded protocol proposed in the Pix4D capture application, but with a flight altitude of 80 m. Furthermore, the flights took place during the day at some point between 8 am and 7 pm.

#### 2.2.2 Ground‐based camera

For data collection, a canon Ixus 180 (20 MP) was used for the landscape scale, while a Campark 4K Ultra HD 20 MP was considered for the national and temporal scales. For the landscape scale, we made videos using a digital camera above the 1 m² subplots. For the national and temporal scales, the cameras were moved above the grass along five 1‐m transects. The camera had a 90° orientation in the direction of the ground. Close to each transect, we had an item with a reference height of 20 cm. For all the data collected, the videos were made at a height of 1 m from the ground.

### 2.3 Structure from motion analysis

**FIGURE 3:**
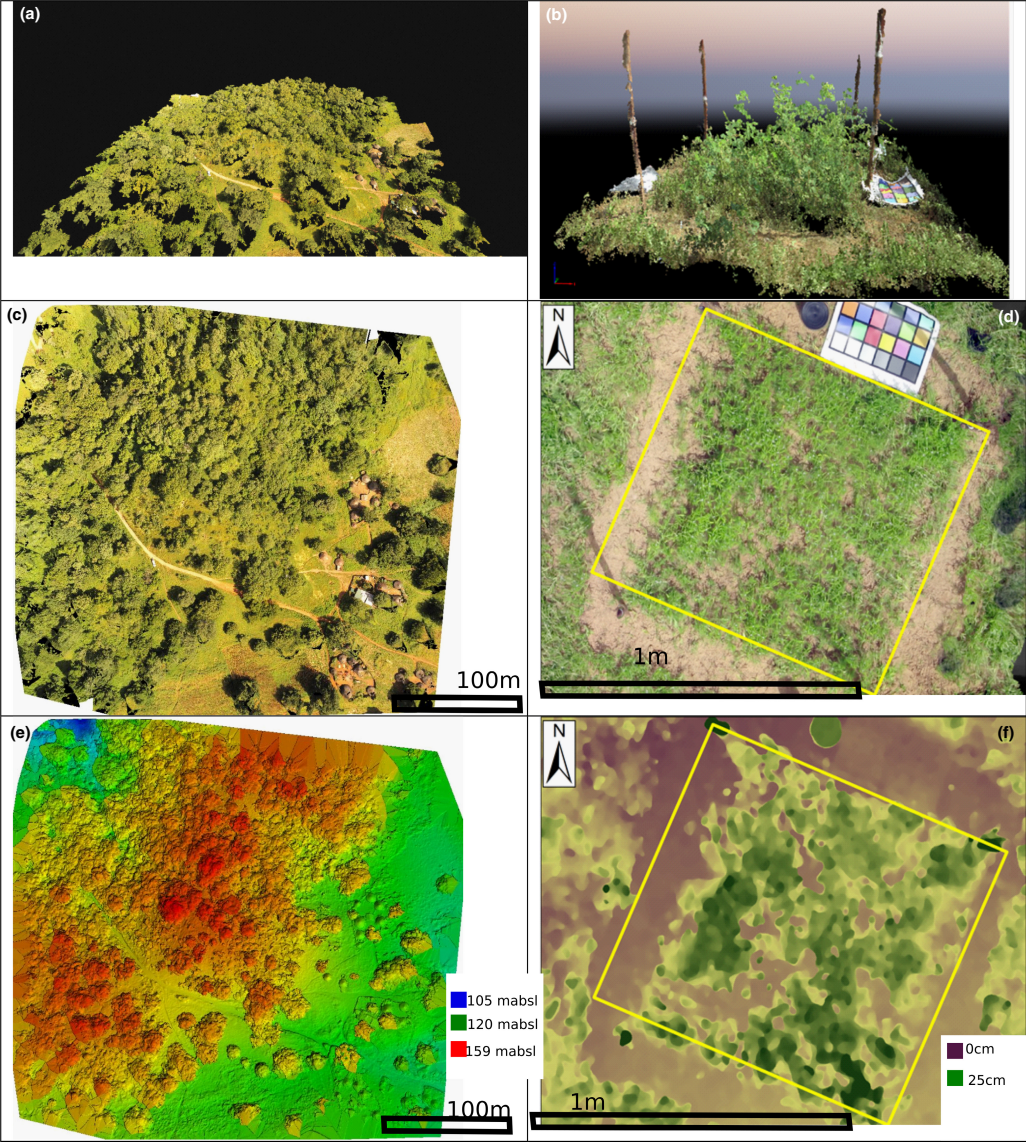
Output from Structure from Motive (SfM) (a) 3D point cloud obtained from the Unmanned Aerial Vehicle (UAV) images, (b) 3D point cloud obtained from the camera images. (c) Orthomosaic obtained from the UAV images, (d) Orthomosaic obtained from the camera images. (e) Digital surface model obtained from the UAV images (The height is in meter above sea level), (f) Digital surface model obtained from the camera images (the height is in cm above ground)

All the images were analyzed with Pix4D software (photogrammetry software) (Figure 3). The videos from the digital camera on the ground were split into images. The split was such that there were between 200 and 300 images per subplot. We used the “3D maps” mode of the software (Figure3a and b), which produces an orthomosaic with a digital number for the red, green, and blue channels (Figure 3c for UAV and Figure 3d for digital camera) and a digital surface model (Figure 3e for UAV and Figure 3g for digital camera). The spatial resolutions were around 3 cm from the UAV images and few mm for the camera. A Digital Terrain Model (DTM) was used for the Landscape UAV (LU) and National UAV (NU) datasets. The Digital Terrain model was an output. The resolution of the DTM was five time the resolution of the other (around 10 cm) For these datasets, HAB height (CHM) was estimated by taking the difference between the DSM and the DTM.

For the outputs based on data from the digital camera on the ground, we extracted the data from the height reference and corrected the DSM of the different plots. From the CHM, we used the average height of all the pixels within the subplot (*H*_mean_) and the maximum height of the subplot (*H*_max_).

Six different indices using red, green, and blue reflectance were calculated (Table 1) (Barbosa et al., 2019; McKinnon & Hoff, 2017). We used the digital numbers when calculating the indices. For the NU dataset, we also computed reflectance using the option in Pix4D software. The indices calculated for reflectance were closely correlated with the indices based on the digital number and we thereby concluded that the digital values could be used directly (Appendix S1).

**TABLE 1:** List of vegetation indices used

Acronym	Definition	Formula	References
NDGRI	Normalized Difference Green Red Index	(R−G)/(R+G)	Lussem et al. (2019)
NDBRI	Normalized Difference Blue Red Index	(B−R)/(B+R)	
NDBGI	Normalized Difference Blue Green Index	(B−G)/(B+G)	
vari	Visible Atmospheric Resistant Index	(G−R)/(G+R−B)	McKinnon and Hoff (2017)
Exg	Excess of green	G−0.39*R−0.61*B	Barbosa et al. (2019)
Gli	Green Leaf Index	(2*G−R−B)/(2*G+R+B)	Barbosa et al. (2019)

### 2.4 Collection of herbaceous aboveground biomass

Herbaceous aboveground biomass (HAB) was collected for each of the 1‐m^2^ subplots. The herbaceous vegetation was cut at soil level, as by Levang & Grouzis (1980). The fresh mass was weighed directly on site. Two different methods were used to estimate dry mass. For the temporal dataset, the whole‐fresh sample was dried at 56°C and the mass was weighed daily. When the samples stopped decreasing in weight, we considered that weight as the dry mass. For the HAB data collected on the landscape and national scales, the large number of samples made it impossible to dry all the samples. We took a composite sample from each site with a known fresh weight, which we dried to obtain the dry matter content. We then applied the dry matter content found for the samples of fresh mass to obtain the dry mass of each sample (Diouf et al., 2015; Ndiaye et al., 2015).

The number of field measurement and the statistic of the fresh and dry mass across the different dataset is in Table 2.

**TABLE 2:** Mean, minimum, maximum, and standard deviation of fresh and dry mass for the different datasets

Dataset	*N*	FM				DM			
		Mean	Min	Max	SD	Mean	Min	Max	SD
LC	35	908.71	140	2680	531.27	335.58	74.20	696.80	172.98
LD	345	712.11	0	2680	536.86	255.84	0.00	797.07	178.83
NC	99	528.93	20	2440	418.79	224.01	8.40	1037.00	177.83
ND	86	504.70	20	2440	407.52	212.46	8.40	1037.00	170.60
TC	29	413.34	61	1674	408.69	98.03	18.71	264.00	73.39
TD	65	781.45	240	1540	362.87	197.18	70.00	382.00	83.89

Note

LC refers to landscape camera, NC refers to national camera, TC refers to temporal camera.

### 2.5 Data analysis

#### 2.5.1 Predicting biomass from SfM outputs

For each of the six datasets even for the datasets with a low number of measurements (TC 29 and LC 35), we randomly separated the data into two groups; 2/3 were used for training (calibration dataset) and 1/3 were used for validation (validation dataset). The model calibration was done using a random forest algorithm with the field samples of fresh and dry HAB as response variables and SfM output as explanatory variables. Due to the unbalanced distribution of HAB (more low than high values), we used a root mean square transformation. The predictor variables were the mean and maximum heights (*H*_mean_ and *H*_max_), the red, green, and blue digital numbers, and the six vegetation indices. Each random forest used 500 regression trees, each time only including six randomly selected predictor variables from one‐third of the calibration dataset. The percentage of explained variance was obtained by cross‐validation against the remaining 33% of the calibration dataset within the random forest. The importance of each predictor variable was estimated by taking the difference in prediction quality based on the mean square error when the randomly selected variable was included and not included in the regression trees. This difference was then averaged over all trees and normalized by its standard deviation.

The calibrated random forest models were used to predict fresh and dry HAB for the validation sites and compared these predictions to the field‐observed HAB of the validation dataset. The agreements between model predictions and field observations were assessed using the root mean squared error (RMSE) and the relative RMSE obtained by dividing the RMSE by the mean of the measured values (RMSE_R_). We also calculated the root median squared error (RMdSE) and the relative root median squared error (RMdSE_R_).

#### 2.5.2 Comparing Camera and UAV output

The goal here was to compare Camera and UAV predictions on the same set of data for subplots where both UAV and camera images were taken. This was the case for 35 subplots of the landscape camera data and also for 81 subplots of the national dataset (NC and NU). A variance partition was made for fresh and dry mass to see how much of the explained variances was similar between the two different estimates, or to what extent they represented complementary sources of information. The variance partition was made using the vegan packages in R. Variance partitioning is a technique that separates the variances of two (or more) sets of variables evaluating the variance explained by the two sets of variables, or only by one set of variables (Borcard et al., 1992; Legendre & Legendre, 2012).

## 3 RESULTS

### 3.1 Using SfM to monitor the change in HAB on a temporal scale

#### 3.1.1 Variability of the growing season HAB according to the digital camera on the ground

On average, HAB was 413.4 ± 408.7 g m^−^² for fresh mass and 98.03 ± 73.39 g m^−^² for dry mass (Table 2). For the fresh mass, the percentage of variance explained by the random forest model was 15.89% with *H*_mean_, *H*_max_, and the VARI index as the three most important variables determining HAB dynamics during then growing season. The *R*² on the validation dataset (10 plots) was 0.8 with a RMSE of 22.48 g m^−^² (RMSE_R_ = 0.06) and a RMdSE of 14.6 g m^−^² (RMdSE_R_ = 0.07%) (Table 3).

For the dry mass, the percentage of variance explained by the random forest model was 41.69% with the same order of importance for the variables as for fresh mass. The *R*² on the validation dataset was 0.79 with a RMSE of 22 g m^−^² (RMSE_R_ = 0.22) and a RMdSE of 13.87 g m^−^² (RMdSE_R_ = 0.18).

#### 3.1.2 Variability of the growing season HAB according to UAV data

The average fresh mass was 781.45 ± 362. 87 g m^−^² and the average dry mass was 98.03 ± 73.39 g m^−^² (Table 2). The random forest model explained 76.99% of the variances of fresh mass and 78.91% of the variance of dry mass (Table 3). The three most important variables were Gli, Exg, and Vari for fresh mass, and Gli, Vari, and H_mean_ for dry mass. The validation indicated an *R* ^2^ of 0.78 for fresh mass (RMSE = 132 g m^−^², RMSE_R_ = 0.16) and 0.27 for dry mass (RMSE = 34 g m^−^² and RMSE_R_ = 0.16) (Table 3).

**TABLE 3:** Results of the random forest models and the different validation indicators

	FM							DM						
	RF	Variable	*R*² test	RMSE (g m^−^²)	RMSER (g m^−^²)	RMdSE (g m^−^²)	RMdSE_R_ (g m^−^²)	RF	Variable	*R*² test	RMSE (g m^−^²)	RMSER (g m^−^²)	RMdSE (g m^−^²)	RMdSE_R_ (g m^−^²)
ALL	71.51	Gli,exg, Vari	0.71	175	0.27	104	0.21	64.64	GLI, exg, Vari	0.53	79.74	0.34	50.31	0.28
Drone	78.65	Gli,, Vari, exg	0.76	153	0.24	112	0.20	68.09	Gli,, Vari, exg	0.73	58	0.24	41.45	0.19
camera	47.81	Gli,exg, Hmean	0.73	166	0.30	106	0.25	35.58	Hmean, Gli, Vari	0.76	74	0.35	48.89	0.28
LU	82.65	Exg, Vari, Gli	0.77	153	0.21	81	0.13	73.82	Exg,,vari, Gli	0.73	59.35	0.23	41.45	0.18
LC	44.03	Vari, Hmean,red	0.65	203	0.21	124	0.16	14.19	Hmean, vari red	0.54	95.2	0.26	72.94	0.23
NU	64.76	Gli, Hmax, HM	0.60	114.15	0.21	119	0.31	49.49	Gli, exg,HM	0.65	62.2	0.29	59	0.31
NC	52.59	exg, Gli, Vari	0.64	153	0.34	102	0.28	38.64	Hmean, exg, Vari,	0.56	72.5	0.38	48.5	0.27
TU	76.99	Gli,exg, Vari	0.78	132	0.16	100	0.14	78.91	Gli,vari, Hm	0.72	34	0.16	20.1	0.11
TC	15.89	Hmean, Hmax,vari	0.80	22.48	0.06	14.67	0.07	41.69	Hmean,HM,vari	0.79	22	0.22	13.87	0.18

Note

RF: percentage of variation explained by the random forest model on the calibration dataset, variable: the three most important variables from the random forest model, *R*² test: *R*² of the model between predicted and measured values based on the validation dataset, RMSE, RMSE_R_, RMdSE, and RMdSE_R_. The left side presents the results for fresh mass and the right side for dry mass. Each line represents the dataset or combination of the datasets used. LU refers to landscape Unmanned Aerial Vehicle(UAV), TU refers to temporal UAV, NU refers to national UAV, TC refers to temporal camera, LC refers to landscape camera and NC refers to national camera.

### 3.2 Using SfM to assess HAB variability across a landscape

#### 3.2.1 Landscape camera results

The landscape camera results based on 35 plots showed that the average fresh mass was 908.71 ± 531.27 g m^−^² and the average dry mass was 333.558 ± 172.998 g m^−^² (Table 2). The random forest model was run on 23 plots of the calibration dataset, and it showed a percentage of explained variance of 44.03% for fresh mass and 14.19% for dry mass. The most important variables were Vari, *H*_mean_, and red for fresh mass, and *H*_mean_, Vari, and red for dry mass. On the validation dataset (12 plots), the *R*² was 0.65 for fresh mass and 0.54 for dry mass with a RSME value of 203 g m^−^² (RMSE_R_ = 0.21) and 95.2 g m^−^² (RMSE_R_ = 0.26), respectively.

#### 3.2.2 Landscape UAV results

For the landscape UAV dataset, the average fresh mass was 712.11 ± 536.86 g m^−^² and the dry mass was 255.84 ± 178.83 g m^−^² (Table 2).

The random forest model was run on 230 subplots and the percentage of variance was 82.65% for fresh mass and 73.82% for dry mass (Table 3). For both analyses, the three most important variables were Exg, Vari, and Gli. On the 115 validation plots, the *R*² values were 0.77 for fresh mass and 0.73 for dry mass. The RMSE for fresh mass was 153 g m^−^² (RMSE_R_ of 0.21) and 59.53 g m^−^² (RMSE_R_ of 0.23) for dry mass.

### 3.3 Using SfM to assess HAB variability on a national scale

#### 3.3.1 National camera results

For the national camera dataset (*N* = 99), the average fresh mass was 528.93 ± 418.78 g m^−^² and the average dry mass was 224.01 ± 177.83 g m^−^². The random forest was run on 66 plots of the calibration dataset. The percentage of variance was 52.59% for fresh mass and 38.64% for dry mass. The three most important variables were Exg, Gli, and Vari for fresh mass, and HM, Exg, and Vari for dry mass (Table 3). The *R*² on the 33 plots of the validation dataset was 0.64 for fresh mass and 0.56 for dry mass. The RMSE was 153 g m^−^² (RMSE_R_ = 0.21) for fresh mass and 72.5 g m^−^² (RMSE_R_ = 0.38) for dry mass.

#### 3.3.2 National UAV results

On the 86 plots representing the national UAV results, the average fresh mass was 504.70 ± 407.25 g m^−^². For dry mass, the average was 212.46 ± 170.60 g m^−^². The random forest was run on 57 plots. The percentage of variance explained was 64.76% and 49.49% for the fresh and dry mass, respectively. The important variables were Exg, Gli, and Vari for fresh mass, and *H*_mean_ exg and Vari for dry mass. The *R*² was 0.64 and 0.56 for fresh and dry mass, respectively. We obtained a RMSE of 114 g m^−^² (RMSE_R_ = 0.21) for fresh mass and 62.2 g m^−2^ (RMSE_R_ = 0.29) for dry mass.

### 3.4 Results on the combination of datasets

#### 3.4.1 Combining all datasets

The random forest was run on the grouping of the six calibration datasets (436 plots). For fresh mass, the random forest explained 71.51% of the variance for fresh mass and 64.64% of the variance for dry mass. For both analyses, the three most important variables were the Gli, Exg, and Vari indices.

**FIGURE 4:**
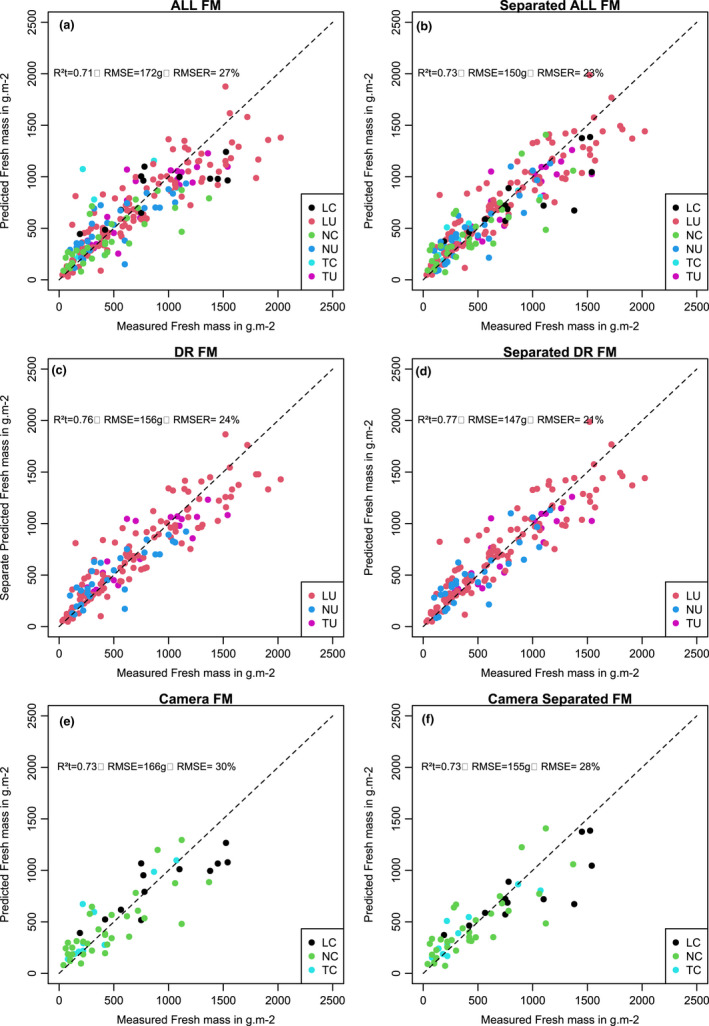
Predicted and measured fresh mass based on the validation dataset. (a) Predicted fresh mass obtained from the random forest model based on combining all the datasets. (b) Predicted fresh mass obtained from the random forest model based on individual datasets separately. (c) Predicted fresh mass obtained from the random forest model based on combining all the drone datasets. (d) Predicted fresh mass obtained from the random forest model based on each drone dataset separately. (e) Predicted fresh mass obtained from the random forest model based on combining all camera datasets. (f) Predicted fresh mass obtained from the random forest model based on each camera dataset separately

On the 220 plots of the validation set, the *R*² was 0.71 with a RMSE of 175 g m^−^² (RMSE_R_ = 0.27). When the random forest model was run on each dataset, the *R*² on the validation dataset was 0.73 with a RMSE of 150 g m^−^² and a RMSE_R_ of 0.23 (Figure 4a and b).

**FIGURE 5:**
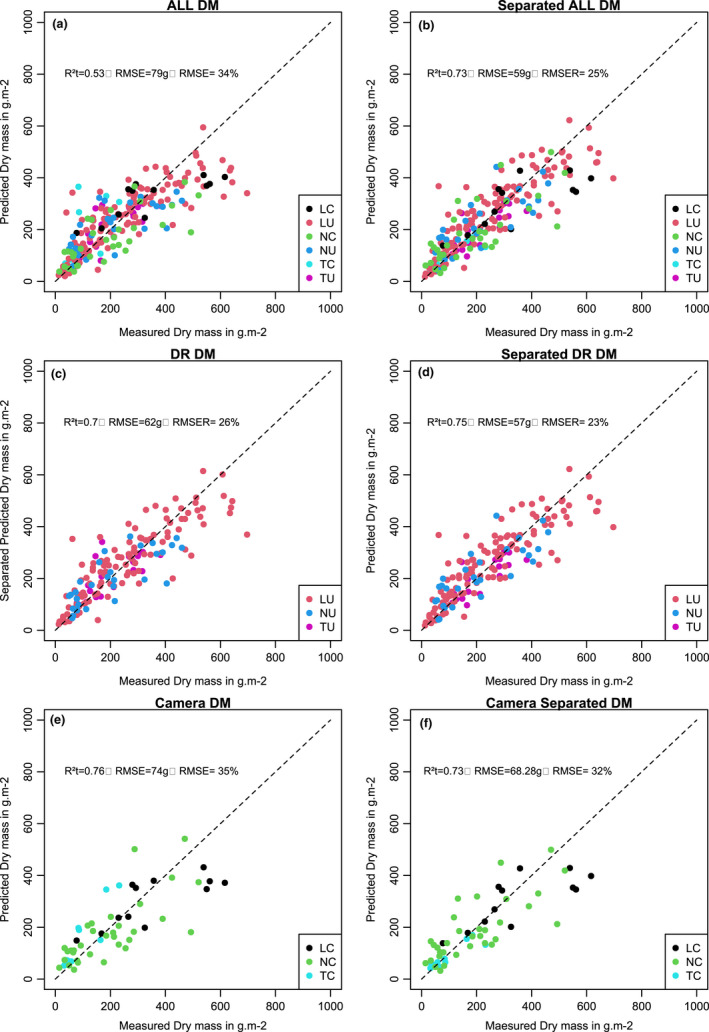
Predicted and measured dry mass based on the validation dataset. (a) Predicted dry mass obtained from the random forest model based on combining all the datasets. (b) Predicted dry mass obtained from the random forest model based on individual datasets separately. (c) Predicted dry mass obtained from the random forest model based on combining all the drone datasets. (d) Predicted dry mass obtained from the random forest model based on each drone dataset separately. (e) Predicted dry mass obtained from the random forest model based on combining all camera datasets. (f) Predicted dry mass obtained from the random forest model based on each camera dataset separately

The *R*² was 0.53 with a RMSE of 79 g m^−2^ (RMSE_R_ = 0.34) (Figure 5a). In comparison, when the random forest model was run on each dataset separately, the *R*² was 0.73 on the validation data with a RMSE of 59 g m^−^² and a RMSE_R_ of 0.25 (Figure 5b).

#### 3.4.2 Combining UAV datasets

The random forest model was run on the combination of the three UAV calibration datasets (i.e., TU, LU, and NU). We obtained a percentage of explained variance of 78.65% for fresh mass and 68.09% for dry mass (Table 2). The most important variables were the same for both fresh and dry mass (i.e., Gli, Vari, Exg).

The relations between predicted values from the random forest made by combining the UAV dataset and the measured values for fresh mass produced an R² on the validation dataset of 0.76 with a RMSE of 156 g m^−2^ (RMSE_R_ = 0.24) (Figure 4c). The R² between the predicted values of fresh mass obtained from the different random forest models run on each dataset separately and the measured values for the UAV datasets was 0.77 with a RMSE of 57 g m^−^² and a RMSE_R_ of 0.23 (Figure 4d). The *R*² of the model built on the combined dataset was 0.73 (with a RMSE of 62 g m^−^² and a RMSE_R_ of 0.24) for dry mass (Figure 4c). For the prediction made on the random forest models constructed separately, the *R*² was 0.73 with a RMSE of 57 g m^−^² and a RMSE_R_ of 0.23 (Figure 5d).

#### 3.4.3 Combining camera datasets

For the model based on all the camera data, the percentage of explained variance for fresh mass was 47.81%, and 35.58% for dry mass. The three most important variables were Gli, Exg, and *H*_mean_ for fresh mass, and *H*_mean_, Gli, and Vari for dry mass.

The *R*² for fresh mass when combining the camera datasets was 0.73 with a RMSE of 166 g m^−^² and RMSE_R_ of 0.30 (Figure 4e). For the same dataset, the *R*² for validation of the model developed on each dataset separately was 0.73 with a RMSE of 68.28 g m^−^², RMSE_R_ of 32% (Figure 4f).

The *R*² for validation of the model when combining all camera data for dry mass (Figure 5E) was 0.76 with a RMSE of 74 g m^−^² (RMSE_R_ = 35%). The *R*² of the model developed on each dataset separately was 0.73 with a RMSE of 68.28 g m^−^² and RMSE_R_ 32% (Figure 5f).

### 3.5 Comparison of camera and UAV data

**FIGURE 6:**
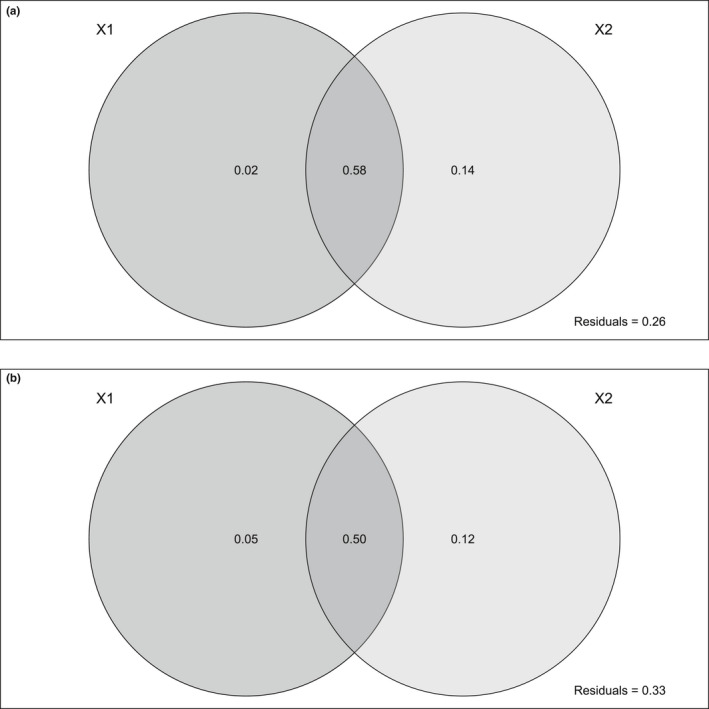
Variance partitioning between Camera and Unmanned Aerial Vehicle (UAV) variables. The darker gray circle on the left (X1) represents the percentage of variance only explained by UAV and (X2) the lighter gray the percentage explained only by the camera variables. The intersection represents the percentage of variance explained by both UAV and camera variables. (a) variance partitioning for fresh mass, (b) variance partitioning for dry mass

For the variance partitioning of fresh mass (Figure 6a), 58% of the variance was explained by both types of data, 14% only by the camera and 2% only by the UAV. The residuals (unexplained variances) amounted to 26%. For variance partitioning for dry mass with 33% of residuals (Figure 6b), 50% of the variance was explained by both types of data, 12% only by the camera and 5% only by the UAV.

## 4 DISCUSSION

### 4.1 Assessing herbaceous aboveground biomass using SfM outputs based on RGB

Our results showed that Structure from Motion (SfM) data obtained from RGB images can be used to assess HAB of the herbaceous layer with relatively good accuracy. For fresh mass, we had errors of around 150 g m^−^² across the different datasets (around 25% of relative error), whereas for dry mass, the errors were around 60 g m^−^². However, there is some room left for improvement to be discussed below. SfM outputs are from two types of information: color indices (Table 1) and height variables derived from the DSM and DTM. Regarding the most important variables, for the color indices the color index based on all three colors (Gli, Exg, Vari indices) proved especially useful. At least one of these variables proved to be among the three most important variables in the 24 models we tested. The green leaf index (Gli) was amongst the three most important variables in 13 models tested and the most important variable in eight models. This is supported by the literature, where the same results were found (Bendig et al., 2014; Lussem et al., 2018; Possoch et al., 2016). For most of the datasets produced, the images were taken at different times of the day and therefore with different light conditions. A color grid was used as part of four of the six datasets, but the correction made with these color references proved to induce more errors at the end (results not shown) and therefore this preprocessing step was omitted from the results presented here. Furthermore, the index based on reflectance data was found to be less related to field measured data than indices based on digital numbers. This result shows that the color indices were related to biomass. Here, we only based our work on RGB images of course, but many studies have used near infrared. (Candiago et al., 2015; Wijesingha et al., 2020). Indeed, it is well known that Infrared‐based indices are linked with the vegetation (Rouse et al., 1974). Increasing the spectral resolution of UAV captors could also be a solution to increase the prediction. However, the cost of a NIR UAV or camera is high, but it would be interesting to compare RGB indices and NIR indices.

The height variables (maximum and mean height of the subplot) obtained from the 3D model were within the top three variables in nine out of 18 models, especially for the dry matter model (six out nine models). The height variables were more important in the models based on camera outputs. Spatial resolution was found to be quite different between the two tools, whereas for the UAV the ground surface distance (GSD) was around 3 cm (a few mm for the camera). The height of the grass in the Sahel region is generally around 40 to 50 cm at maximum vegetation growth (Boudet, 1984). GSD may not be precise enough for the height of grass and its variation within the natural vegetation. In other studies, using UAVs on grasslands, the variables from the 3D model were found to be more predictive (Lussem et al., 2018, 2019). In some studies, the measurements were taken on management experiments that created high variability in grass heights between the different plots. Their flight height was lower than in this study. We chose a height of around 80 m due to the presence of trees. It was not that the height of the tree could directly impede a flight altitude of 20 m (the highest tree was around 15 m tall), but because the 3D model of the trees would have been sub‐optimal and may have impeded the creation of the mosaics. Furthermore, we also used the Landscape and National scale images to work on tree communities (Bossoukpe, Faye, et al., 2021). For the camera, an area of bare soil was close to the subplot. Indeed, we cut the grass close to the subplot. This bare soil created a difference in height variability and the spatial resolution was much better. For the National and some of the Temporal datasets, we measured the height of several herbaceous individuals in the subplot. The mean heights measured from the field were compared to the mean heights obtained from the 3D model for the camera. (*R*² = 0.33, results not shown).

Errors could also come from the manual measurements of the herbaceous vegetation (destructive sampling). For example, the percentage of variance explained was generally higher for fresh mass than for dry mass, except for the analysis covering the temporal scale. One reason might be that in the datasets of the temporal scale analysis the dry mass was measured for all samples. For the other four datasets, the dry matter content was only measured for a composite sample. The composite sample may have created some bias in the measurement (Diouf et al., 2015).

One problem is also the positioning of the measurements, especially for the UAV. We used markers on the soil (wooden triangle or plastic bag), but some inaccuracies still remained. Ideally, it would be better to have a 1 m² subplot with a visible border.

### 4.2 Comparing camera and UAV tools

We investigated using Structure from Motion (SfM) from two different types of sensor systems: Unmanned Aerial Vehicle (UAV) and a ground digital camera. Overall, we found higher percentages of explained variance for the UAV than for the camera. However, when we compared the same set of subplots for both tools (Figure 5), the camera outputs were slightly better than those of the UAV. We surmise that the difference observed between the two tools was due to a better assessment of vegetation height by the camera.

Although both tools are based on the same concept, they have quite different uses. UAVs are now frequently used for vegetation monitoring. Our work confirmed that UAVs, especially when equipped with a low‐cost RGB sensor, can be used to adequately quantify the phytomass of herbaceous layers in a savannah ecosystem. UAVs can be used to map areas from 1 ha up to 20–30 ha (potential area with one UAV battery) depending on the flight plan and UAV characteristics. The spatial scale of this approach is well suited to experiments on permanent grasslands (Lussem et al., 2019). For many temperate ranching systems, UAVs could also be used to assess biomass on a plot scale to evaluate the available livestock feed. However, in Sahelian countries, as in many pastoralism areas, rangelands are used as a common resource exploited by several farmers across large areas. UAVs could be used as an intermediate tool to close the spatial gap between field observations and satellite images. Many UAV studies have shown that woody vegetation can also be characterized using UAVs (Bossoukpe, Faye, et al., 2021; Mayr et al., 2018). Trees are an important part of the savannah ecosystem, even with their low density. One interesting feature of the UAV tool is the ability to evaluate both strata of vegetation (Bossoukpe, Ndiaye, et al., 2021). It can also be used to assess the spatial structure of vegetation, to show the effect of trees on the herbaceous layers, for example.

UAV use does have some limitations. The spatial resolution (around 3 cm) of the UAV is not high enough to identify different individual grasses, so it remains a tool for describing overall vegetation. Furthermore, UAV systems cannot monitor grasses that grow underneath tree crowns. The herbaceous vegetation is indeed influenced by the presence of trees, and the vegetation under trees often has a different botanical composition and phytomass. In Savannahs with a low tree cover (~5%), the impact of trees is limited. However, in denser savannah systems, evaluation of the herbaceous phytomass of the ecosystem based only on the herbaceous layers outside the tree cover may be more problematic, and in forest areas of closed canopies RGB UAV‐based monitoring of the understory is, of course, not possible. Another limitation of the use of UAVs relates to legislation issues (Haula & Agbozo, 2020). Indeed, the use of UAV technology is limited by law. In many countries, some areas are restricted, such as populated areas, airports, or other sensitive areas (e.g., military) and for some countries the use of UAVs is totally forbidden and/or detailed legislation has not effectively been implemented. Furthermore, UAV licenses are required in many countries, which means that UAVs cannot be used by just anyone, and that potential users will have to take training courses to obtain the license.

Using SfM approaches with a simple digital camera has been much less studied than with UAVs (Andújar et al., 2018; Cooper et al., 2017), even though it is easier and cheaper to use a camera. In the study by Cooper et al. (2017), SfM was obtained from photography and here we used a video to simplify the field work and to be able to subsequently select the number of images implemented in PIX4D software.

The digital camera has advantages for use on a fine spatial scale, due to its higher definition (a few mm as opposed to 3 cm) as compared to UAV systems. In cases of low phytomass, such as early in the growing season, or for very low and sparse herbaceous layers, individual grasses can be easily identified (Figure 1). Structure from Motion has already been used on individual plants (An et al., 2017; Andújar et al., 2018) and we believe that other variables from the herbaceous stratum could be assessed (individual height, leaf area, species recognition, etc.). However, for dense herbaceous vegetation, it is impossible to identify individuals. In homogeneous cover, the Structure from Motion process can be challenging, with lower applicability. In such cases, only variables on a community scale can be assessed, such as the phytomass or the mean height of the layer. (Bossoukpe et al., 2020).

Another interesting feature of the camera approach is its ease of use, and the tool can be used to develop a herbaceous growth monitoring programs across a large scale (national scale). In Ireland, a participatory monitoring program has been in operation for several years, where farmers across the country estimate grass growth (Hanrahan et al., 2017). The data are available online and can be used by farmers or the authorities. In Sahelian countries, this kind of data could greatly enhance the current monitoring framework (Diouf et al., 2015).

However, compared to UAVs, using a digital camera has several drawbacks. Of course, a digital camera cannot be used to produce maps and the orthomosaic is not geotagged. Moreover, a height reference is needed. For the UAV, several subplots can be taken for a single flight, whereas for the camera each sample requires an SfM process and the number of pix4D projects needed for calibration will be larger (one per sample).

### 4.3 Using the SfM methodology across spatio‐temporal scales

Our results showed that Structure from Motion (SfM) can be used on the three different scales tested here. Regarding the different scales and datasets, the range of the biomass masses was quite similar between the different datasets (Table 2). Between the six datasets, the percentage of variance was clearly higher for the datasets including more measurements. All in all, we observed similar errors across the three different scales and showed that SfM can be used in all instances of scales.

On the temporal scale, we tested SfM use during the growing season. The growing season in the Sahel region is very short and erratic during the few months of the wet season and UAVs and cameras can be used to monitor grass growth with a high measurement frequency. On an experimental design with differences in management or water input on small plots, such as (Cisse, 1986; Hiernaux & Hérault, 2003; Hiernaux et al., 1995; Hiernaux & Turner, 1996), SfM could be used to monitor the dynamics of the herbaceous layers indirectly with high frequency.

Here, we only tested the method during the wet season and on green grass. However, dry grass during the nine months of the dry season is an important source of animal feed in the region. The continuous monitoring of this remaining dry biomass is key for livestock management in pastoral systems. Remote sensing work based on the soil tillage index using shortwave infrared information has been carried out to evaluate dry biomass (Kergoat et al., 2015). The SfM methodology could be a complementary solution for estimating the volume of dry grass resources based on the soil cover. Some preliminary work has been undertaken using cameras combined with growing season data (Diedhiou et al., 2021).

Structure from Motion can also be used to assess the spatial variation of biomass in the herbaceous stratum. Here, we tested the two tools on two different spatial scales: the landscape scale, where all the measurements were taken on a research station of around 6800 ha in Dahra Djoloff. The variability of herbaceous biomass on this scale is driven by the microtopography that reflects differences in soil types and impacts water availability for the herbaceous biomass. Trees also influence the herbaceous layers and management of the different plots (free grazing, enclosure, and dense *Senegalia senegalensis* planting). Here, SfM tools can be used to test the impact of these factors on the herbaceous layers (Diatta et al., 2021), especially with a focus on the temporal aspect (within or between growing seasons). For more applied and operational usage, operations on a landscape scale should be considered in relation to the management type and size involved. In the Sahel, ranching farms are not really frequent, but SfM, especially based on UAVs, could be an interesting tool for evaluating grass availability in different plots. In pastoralism systems, landscape‐scale monitoring could be of interest for some actors. For example, pastoral units with collective management are installed in the different Sahelian countries. UAVs could be useful for monitoring the different rangelands within the pastoral unit.

Our analysis of monitoring on a national scale involved a dataset collected in the northern and southeastern parts of Senegal. In addition to selecting plots based on topography and management type, the plots were located along a rainfall gradient (from an average of 200 mm per year to more than 1000 mm per year), and a soil type gradient from sandy soils to ferralitic soils was also considered. A national scale monitoring program is important for livestock policy, and indeed in several Sahelian countries the available biomass (herbaceous layers and foliage of woody species) is monitored on a national scale taking a sample‐based approach. The aim is to evaluate the national fodder balance and eventually develop local and national policies (pastoral mobility, distribution of animal feeds, etc.) (Touré & Ickowicz, 2014). Our results showed that SfM tools could be used across Senegal and possibly other Sahelian countries. For the UAV approach, one limitation is the southernmost region where the tree cover can be dense, and only the grass biomass outside the tree cover can be monitored.

UAVs or other very high resolution (VHR) remote sensing systems could be used to simplify scaling‐up from field measurements and conventional remote sensing systems (Taugourdeau et al., 2014). Indeed, a hybrid model combining fewer measurements could replace actual field measurement programs that require an extensive protocol to assess the heterogeneity of the biomass layer, as already proposed for other communities (Kattenborn et al., 2019).

The camera approach could also be used on a national scale. One interesting feature of a camera monitoring protocol is that it requires few qualifications and equipment and one way forward will be to use this tool as part of a participatory observatory. Different observers across the country could take RGB images using a digital camera/smartphone, which would be analyzed to monitor biomass across the country.

### 4.4 Combining the use of datasets

We obtained better results for biomass estimation when more sampling data were used. One way forward would be to combine several datasets to build a large calibration dataset. However, our results showed that prediction was not better and generally performed slightly worse when our calibration was based on a combination of several datasets, as compared to when calibration was based on a single dataset (Figures 3 and 4). This result was found even for the dataset with few observations, such as the "Temporal Camera" (29 plots) and the "Landscape Camera" (35 plot) approaches. For these two datasets, the random forest and the separation in calibration set and validation set are necessarily adapted due to the low number of measures but we used the same methodology for all datasets.

For both, the percentage of variance of the random forest was quite low, but the prediction on the validation dataset was not markedly different from the other datasets. The difference between the combined datasets and single datasets was less pronounced when the combination of datasets was made between UAV and camera, separately. The small difference between the results based on calibration within a dataset and calibration made by combining the datasets from the same tool showed that tool‐specific calibrations were quite generic. It can be hypothesized that the calibrations developed in this work could be used directly on new datasets of each type. We found small differences between the makes of UAVs (Spark or Anafi) and cameras used (Campspark and Canon) and on the image acquisition protocols (altitude, type of flight plan).

It would be interesting to produce more data using UAVs on herbaceous plants involving different types of vegetation and protocols. The goal of such work would be to further test the robustness and generalness of UAV biomass relations for African savannahs.

## 5 CONCLUSION

We showed that RGB images used for structure from Motion (SFM) can be used to assess aboveground biomass of the herbaceous layers in a Sahelian savannah ecosystem. We tested SfM processing for biomass estimates using data from Unmanned Aerial Vehicles (UAV) and ground digital cameras and on three scales. The three different scales included spatial scales within a landscape or across a country and, lastly, the temporal scale measuring continuously over the growing season. Our results showed that this approach could be adequately used on all the different scales and testing of the methods also took into consideration variations in land management and soil types for a rainfall gradient from arid to sub‐humid conditions. The ground digital camera data were found to be slightly more accurate than the UAV data, but UAV measurements have the advantage of facilitating the scaling‐up of field measurements to areas of several ha. Herbaceous aboveground biomass mapping by SfM can be used to undertake studies of ecological processes at a high spatial resolution and provides an image source building an important bridge in spatial scales between field observations and satellite remote sensing images.

## CONFLICT OF INTEREST

The authors declare no conflict of interest.

## AUTHOR CONTRIBUTIONS

**Simon Taugourdeau:**Conceptualization (lead); Data curation (lead); Formal analysis (lead); Funding acquisition (equal); Investigation (equal); Methodology (lead); Validation (lead); Visualization (lead); Writing – original draft (lead). **Antoine Diedhiou:** Investigation (equal); Software (equal). **Cofélas Fassinou:** Formal analysis (equal); Investigation (equal). **Marina Bossoukpe:** Conceptualization (equal); Data curation (equal); Investigation (equal). **Ousmane Diatta:** Investigation (equal); Methodology (equal). **Ange N'Goran:** Investigation (supporting). **Alain Audebert:** Writing – review & editing (supporting). **Ousmane Ndiaye:** Supervision (supporting); Writing – review & editing (supporting). **Abdoul Aziz Diouf:** Writing – review & editing (supporting). **Torbern Tagesson:** Writing – review & editing (equal). **Rasmus Fensholt:** Writing – review & editing (equal). **Emile Faye:** Conceptualization (equal); Methodology (equal).

### OPEN RESEARCH BADGES

This article has earned an Open Data Badge for making publicly available the digitally‐shareable data necessary to reproduce the reported results. The data is available at https://doi.org/10.5281/zenodo.6421543, https://doi.org/10.5281/zenodo.5148337and https://doi.org/10.5281/zenodo.5145395.

## Supporting information

Appendix S1

## Data Availability

The DOI of National UAV dataset is 10.5281/zenodo.5148337. The DOI of Landscape UAV dataset is 10.5281/zenodo.5145395. The DOI of Temporal UAV dataset is 10.5281/zenodo.6421543.
