# Supplementary material for: Estimating herbaceous aboveground biomass in Sahelian rangelands using Structure from Motion data collected on the ground and by UAV
Source: Ecol Evol. 2022 May 1;12(5):e8867. doi: 10.1002/ece3.8867 (PMC9057245; doi:10.1002/ece3.8867)
Supplement: Supplementary file 1 — Appendix S1 [file ECE3-12-e8867-s001.docx]

Supplementary

Corrected data versus uncorrected

Supplementary results, Digital number - Reflectance.

In the orthomosaic, the colors are encoded in each pixel in digital number format. In pix4D mapper software, reflectance can be calculated from the digital number. On the National UAV dataset, we computed the reflectance in pix4d software and computed the different indices with both the digital number and reflectance; we carried out a PCA with the two sets of variables.


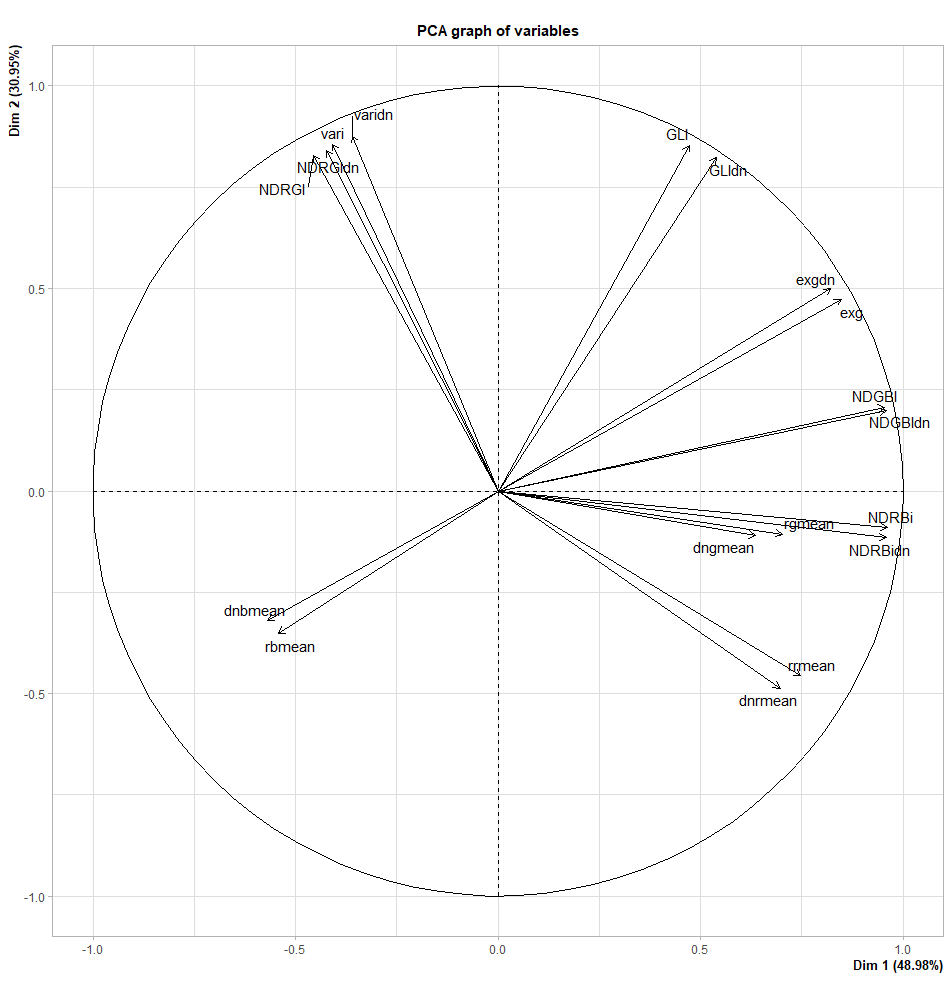


Our prediction was based on the random forest analysis with either color index based on the digital number or reflectance. We obtained 67.37% of explained variance with the digital number and 60.74 with reflectance for fresh mass, and 57.79% and 52.13% respectively for dry mass.

With the lower variance explained and the fact that reflectance calculation induces more calculation time, we chose to use the digital number.
